# Home-Based Care Management for Patients Post-Heart Failure Index Hospitalization: A Comprehensive Review

**DOI:** 10.7759/cureus.83017

**Published:** 2025-04-25

**Authors:** Prafulla Kerkar, Sivadasanpillai Harikrishnan, JP S Sawhney, Aditya Kapoor, Chetan Gharat

**Affiliations:** 1 Interventional Cardiology, Asian Heart Institute, Mumbai, IND; 2 Cardiology, Sree Chitra Tirunal Institute for Medical Sciences and Technology, Trivandrum, IND; 3 Cardiology, Sir Ganga Ram Hospital, Delhi, IND; 4 Cardiology, Sanjay Gandhi Postgraduate Institute of Medical Sciences (SGPGIMS), Lucknow, IND; 5 Medical Affairs, Lupin Digital Health, Mumbai, IND

**Keywords:** care pathway, guideline-directed medical therapy, patient-centered home care, patient education, workbook

## Abstract

Heart failure (HF) is a clinical syndrome characterized by cardiac structural or functional abnormalities, often leading to hospitalization. Common causes of HF include ischemic and rheumatic heart diseases, with a male predominance. Diagnosis relies on clinical evaluation, electrocardiogram, chest X-ray, and biomarkers like N-terminal pro-B-type natriuretic peptide. Left ventricular ejection fraction classifies HF into reduced, mildly reduced, preserved, and improved ejection fraction. Guideline-directed medical therapy includes angiotensin-converting enzyme inhibitors, beta-blockers, mineralocorticoid receptor antagonists, and sodium-glucose cotransporter-2 inhibitors. Surgical interventions, such as coronary artery bypass grafting, device implantation, heart transplantation, and percutaneous coronary intervention, are also employed. Emphasis on multidisciplinary management, patient education, and lifestyle changes improves adherence and reduces hospital readmission rates. Post-hospitalization management increasingly relies on home-based care, achieving outcomes equivalent to clinic-based programs, including improved quality of life, lower mortality, and reduced healthcare costs. The integration of telehealth and mobile applications has expanded remote monitoring and patient engagement. However, challenges such as limited infrastructure, resource constraints, and the lack of standardized protocols hinder optimal implementation. This review highlights the essential role of home-based care for patients with HF post-hospitalization and the need for a comprehensive workbook with a care pathway that provides information on medications, follow-up, and dietary guidelines. The workbook also equips the cardiovascular care team with the skills and knowledge needed for high-quality, patient-centered, home-based care.

## Introduction and background

Heart failure (HF) is a clinical syndrome and a leading cause of hospitalization [1], characterized by symptoms resulting from structural or functional cardiac abnormalities. This is supported by elevated levels of natriuretic peptides or evidence of pulmonary or systemic congestion [2]. The estimated prevalence of HF in India is approximately 1% of the total population or about 8-10 million individuals [3]. The estimated mortality attributable to HF is about 0.1-0.16 million individuals per year [3].

It is classified based on left ventricular ejection fraction (LVEF) and clinical symptoms [4]. As per expert opinion from India, management of HF supports the use of quadruple therapy by following guideline recommendations from the European Society of Cardiology (ESC) and the American College of Cardiology/American Heart Association/Heart Failure Society of America (ACC/AHA/HFSA) [5].

Multidisciplinary management of patients with HF and regular follow-up are effective in enhancing patient adherence, reducing hospital readmissions, and improving survival. The effects of HF clinics that involve multiple disciplines were separated from programs that offered specialized follow-up in a non-clinic setting. The programs conducted in a home-based setting are equally effective in reducing mortality and rehospitalization compared to those in a clinic setting [1]. Home-based care is managed and monitored by a multidisciplinary team, led by doctors, and involves other licensed healthcare professionals such as nurses and therapists. However, self-monitoring by patients alone has not shown the same level of effectiveness. Home care encompasses services provided either by licensed healthcare professionals or to be undertaken by patients post-operatively at their homes. These home-based care services are provided to adults, seniors, and pediatric clients who need support post-hospitalization or to maintain independence at home, thereby minimizing hospital visits [6]. In the post-COVID-19 era, home-based and digital care has emerged as a new evolutionary tool [7]. The primary aim of home health care continues to be ensuring patients have self or family care while minimizing unplanned hospitalizations. These unplanned hospitalizations can result in complications, elevated morbidity, added stress for both patients and caregivers, and higher costs for providers and payers [8].

This review highlights the essential role of home-based care for patients with HF post-hospitalization. It covers the epidemiology, prognosis, etiology, classification, diagnosis, and treatment strategies for managing HF, with a particular emphasis on the importance of home-based care. The article emphasizes the necessity of a workbook that offers crucial information on medications and dietary guidelines that patients should follow. The workbook also equips members of the cardiovascular care team with the skills and knowledge necessary to provide high-quality, patient-centered care in the home setting.

## Review

Epidemiology, etiology, and prognosis of HF in India

Cardiovascular diseases continue to be a major global source of morbidity and mortality. A significant contributor to the burden of cardiovascular diseases is the presence of disparities in cardiovascular risk factors across different populations. Cardiovascular risk is found to be associated with lower socioeconomic status, including classic factors like education, income, employment, and living conditions. Additionally, food and housing insecurity and insufficient insurance coverage compound the risks. Moreover, there is also the influence of mental health conditions like depression and anxiety on cardiovascular health [9].

In India, patients develop HF at a mean age of 61.2 years as reported in the Trivandrum HF Registry (THFR), 58.9 years in the Medanta Registry, and 56 years in the international congestive HF Indian subgroup. The male-to-female ratio is skewed toward men (70:30 in the THFR and 83:17 in the Medanta Registry), likely due to greater healthcare-seeking behavior among men. Ischemic heart disease is the leading cause of HF in India. However, rheumatic heart disease is also a notable contributor, accounting for 8% of cases in the THFR and 5% in the Medanta Registry. Diabetes mellitus is more prevalent among Indian patients. The in-hospital mortality rate for HF in India is 8.4%. Additionally, the one-year mortality rate for HF in India stands at 37%, according to international congestive HF data [10-14].

Classification of HF

The HF classification is based on LVEF. It includes several categories: HF with reduced ejection fraction (HFrEF), defined as an LVEF of 40% or less; HF with mildly reduced ejection fraction (HFmrEF), which encompasses an LVEF of 41% to 49%; and HF with preserved ejection fraction (HFpEF), characterized by an LVEF of 50% or greater. Additionally, there is HF with improved ejection fraction (HFimpEF), which refers to patients with symptomatic HF and a baseline LVEF of 40% or less who experience a rise of at least 10 percentage points in LVEF, with a subsequent measurement exceeding 40% [4]. 

Diagnosis and treatment strategies for the management of HF

Early diagnosis of HF is important to initiate appropriate treatment; however, diagnosis can be challenging, and hence, clinical evaluation and diagnostic tools are important aspects for accurate diagnosis of HF. Clinical evaluation includes a detailed history and physical examination. Initial investigation includes blood estimation followed by physical examination [15]. Abnormal electrocardiogram (ECG) findings, such as pathological Q waves and T-wave inversions, often indicate myocardial infarction or ischemia, which can lead to left ventricular (LV) dysfunction [16]. The Cornell criteria improve the sensitivity of ECG in detecting LV hypertrophy [17]. A chest X-ray showing cardiomegaly and congestion serves as an indicator of HF. The N-terminal pro-B-type natriuretic peptide (NT-proBNP) levels and 2D echocardiography imaging are essential for diagnosing and phenotyping HF. The LV end-diastolic pressure (LVEDP) is a key indicator of LV diastolic function. Elevated LVEDP suggests HF and can be assessed using various non-invasive echocardiographic parameters. The mitral E-wave to mitral annular velocity ratio (E/e′ ratio) evaluates LV filling pressure [18], and an E/e′ >14 suggests elevated LVEDP [19]. Tricuspid regurgitation velocity (TRvmax) measures the velocity of the tricuspid regurgitation jet and can estimate pulmonary artery pressure, with TRvmax >2.8 m/s indicating a high probability of pulmonary hypertension [20]. The left atrial volume index (LAVI) detects diastolic dysfunction, with LAVI >34 mL/m² indicating persistently elevated LVEDP [21,22]. Heart failure with preserved ejection fraction (HFpEF) presents diagnostic challenges, but several scoring systems and biomarkers have been developed to assist in diagnosis. The H2FPEF and HFA-PEFF score are convenient risk stratification tools for diagnosing HFpEF [23]. The NT-proBNP is a key biomarker in the diagnosis and management of HF [24]. Furthermore, the six-minute walk test can be utilized to evaluate exercise capacity in patients with pulmonary hypertension [25].

Management of HF involves patient education, pharmacological and non-pharmacological strategies, and the implantation of devices. As soon as a diagnosis of HF is made, communication between patients and caregivers becomes essential. The aim of patient education is to improve knowledge about HF, motivate self-care, and provide tips for disease management. Maintaining a proper diet and exercise routine is also a crucial part of the management strategy [15]. Adherence to guideline‐directed medical therapies (GDMTs) has been shown to improve survival in HF patients with reduced and mildly reduced ejection fractions [26]. Pharmacological management includes the use of angiotensin-converting enzyme inhibitors, angiotensin receptor blockers, angiotensin receptor-neprilysin inhibitors, beta-blockers, mineralocorticoid receptor antagonists, diuretics, and sodium-glucose cotransporter 2 (SGLT2) inhibitors (Table 1) [15].

**Table 1:** Treatment recommended for management of HF ARNi, angiotensin receptor-neprilysin inhibitor; HFrEF, heart failure with reduced ejection fraction; HFmrEF, heart failure with midrange ejection fraction; HFpEF, heart failure with preserved ejection fraction; MRAs, mineralocorticoid receptor antagonists; SGLT2i, sodium-glucose cotransporter 2 inhibitor. Adapted from ref [5] under Creative Commons CC-BY-NC license

Drugs	Recommendation
ARNi	HFrEF
	HFmrEF
	HFpEF
Beta-blocker	HFrEF
	HFmrEF
MRAs	HFrEF
	HFmrEF
	HFpEF
SGLT2i	HFrEF
	HFmrEF
	HFpEF

Surgical interventions for HF encompass a range of procedures aimed at improving cardiac function and patient outcomes. They include an implantable cardioverter defibrillator, an LV assist device, coronary artery bypass grafting, heart transplantation, and percutaneous coronary intervention (angioplasty) [27].

According to expert opinion from India, HF management should be tailored to each patient's clinical characteristics, comorbidities, and preferences. The SGLT2 inhibitors are recommended for all individuals with HF who have a high cardiovascular risk, as they have proven effective regardless of ejection fraction [5].

Importance of home-based care post-HF hospitalization

Transitioning patients from hospital to home care necessitates ongoing safety measures, requiring coordinated care from both formal and informal caregivers, ensuring the same level of care as in hospital settings [28]. Cardiac rehabilitation (CR) for managing cardiovascular diseases has yet to become mainstream in India. Research indicates that home-based care can be as effective as center-based CR in addressing these challenges [29]. It is also important to understand the needs of caregivers, as they play a crucial role in assisting with activities of daily living, improving and maintaining self-care, providing physiological support, and helping individuals navigate health systems [30].

**Figure 1:**
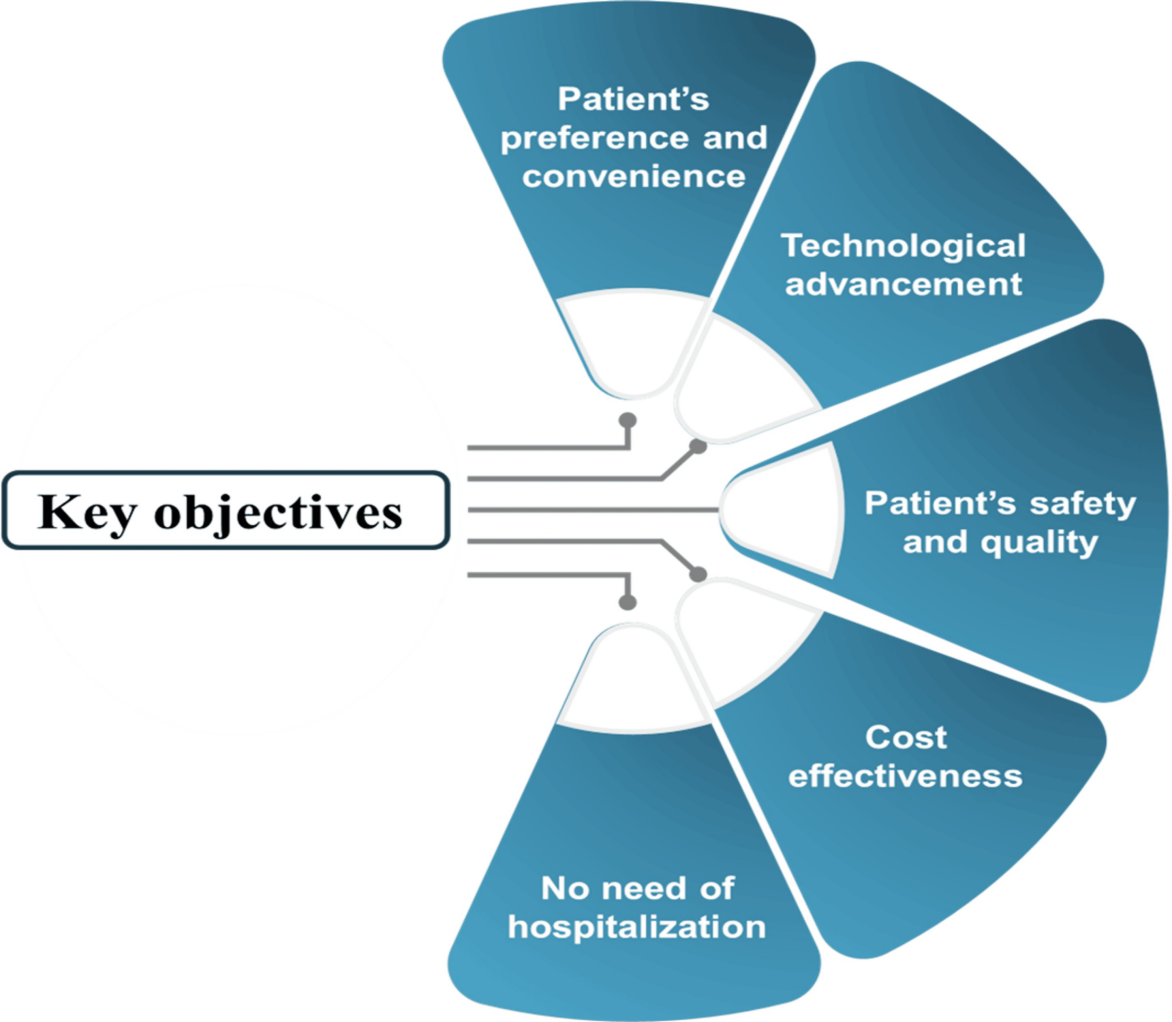
Key objectives for developing home-based care Original figure

The use of mobile health applications in managing HF shows promise for reducing readmissions and healthcare expenditures. In a cross-sectional analytical study, 90 patients diagnosed with HF were discharged from a tertiary care unit, and a tele-interview was conducted. The findings indicated low adherence to physical exercise and weight management among the participants. Notably, more than one-third of the population expressed a willingness to self-record their measurements and utilize mobile applications. The most commonly requested features for these applications included information reminders, health education, chat functions with nurses, physical activity tracking, symptom management, and dietary guidance [31]. A randomized controlled trial assessed the nurse-led home-based HF management program (HOME-N), which featured an HF checklist (Holiday card), weekly telemonitoring of vital signs (blood pressure, heart rate, and weight) using the mobile app ‘Dhadkan’, and telephonic follow-ups over three months. Results showed that the HOME-N program significantly enhanced quality of life and medication adherence among HF patients [32]. Meta-analysis findings indicate that home care is associated with lower all-cause mortality rates, reduced hospitalizations, and emergency department visits, as well as enhanced quality of life and cost savings [33]. The pilot study indicates that an early discharge strategy, supported by home healthcare visits, may be an effective approach to reduce the cost of care for patients with chronic HF [34]. Home-based CR results in short-term improvement in exercise capacity and health-related quality of life for HF compared to usual care and it appears to be safe [35]. The key objective of home-based care is depicted in Figure 1.

Components of home-based care

Cardiac Rehabilitation

CR is a complex intervention with the involvement of several components like exercise training and physical activity promotion, health education for patients, cardiovascular risk management, and psychological support, personalized to the individual needs of patients with heart disease [36]. The Rehabilitation Therapy in Older Acute HF Patients (REHAB-HF) trial evaluated a tailored, progressive rehabilitation intervention targeting four physical-function domains: strength, balance, mobility, and endurance. Each domain had specific interventions such as strength focused on functional strengthening exercises such as sit-to-stand from a chair, side step-ups, stand-to-squat, and open chair strength exercises. Balance interventions included standing balance exercises and stand-and-reach balance exercises. Mobility includes accelerated gait and start-and-stop and turning exercises. Endurance exercise includes walking and activities like the NuStep. The primary outcome was the score on the Short Physical Performance Battery (total scores range from 0 to 12, with lower scores indicating more severe physical dysfunction. This trial with multiple physical-function domains resulted in greater improvement in physical function than usual care [37,38].

Patient Education

Healthcare providers should educate patients about healthy diets and family-based education to promote overall well-being. Patients should be educated on symptom monitoring, dietary recommendations, and lifestyle modifications. A comprehensive management plan should be developed, outlining goals, dietary recommendations, and medication side effects. This equips patients and their families with the knowledge and tools to navigate their health journey confidently and make informed decisions [39].

According to a randomized controlled trial, the mobile app ‘HF-Smart Life’ incorporates educational materials with intuitive pictures and animations, a daily health check-up diary, a question-and-answer feature, and a 1:1 chat. It reported improvements in functional class and cardiac diastolic function. The app also appeared to benefit patient’s continuous self-management and health outcomes, as well as the healthcare provider’s ability to monitor, communicate, and collaborate with patients to achieve better HF treatment [40]. A pilot study, the ‘The Hellenic Educational Self-care and Support HF app (ThessHF app)’ encourages patients to perform self-care steps like measuring weight and blood pressure and assessing potential dyspnea. It also reminded patients to take their medication and provided education through weekly quizzes. The app was associated with an improvement in the quality of self-care quality in patients with HF [41].

Fluid and Diet

Patients with advanced HF should reduce fluid intake based on body weight and limit salt consumption to prevent fluid retention. Patients with fluid overload and those experiencing severe congestive HF should be advised to limit their fluid intake [42]. Alcohol should be avoided, as it causes cardiomyopathy and cirrhosis [43]. Caution is needed with salt substitutes due to potential potassium content, especially if on RAAS blockers. In India, for severe cases, salt intake is initially limited to one-fourth teaspoon per day (500 mg/day). Afterward, it can be gradually increased to half to three-fourths of a teaspoon (2000-3000 mg/day) with the use of diuretics [42]. Additionally, maintaining a healthy weight and avoiding overeating is crucial for overall heart health [15,43]. The National Institute of Nutrition recommends an intake of folic acid, and dietary fiber, along with moderate use) of edible oils. For adults with a sedentary lifestyle, the recommended visible fat intake is 25 g/day, while those involved in hard physical work should consume 30-40g/day. In the Indian context, edible oils such as mustard and rapeseed oil, due to low saturated fatty acid, and high monounsaturated fatty acid content, are advised. Blends of edible oils like rice bran and safflower oil, coconut and sesame oil, and canola and flaxseed oil are recommended to help reduce the risk of coronary heart disease, followed by ghee/butter/vanaspati for the management of heart disease. Also, excessive consumption (>200 mg) of caffeine is known to cause negative effects like anxiety and nervousness additionally increasing blood pressure and cause abnormalities in the heartbeat; thus one cup of coffee for both women and men is recommended [43,44]. The Mediterranean diet, consisting of fish, whole grains, fruits, unsaturated fats, vegetables, legumes, and nuts, has been shown to reduce cardiovascular morbidity and mortality [45,46]. Other diets like the DASH diet (rich in fruits and vegetables, low-fat dairy, whole grain, poultry, fish, nuts, and seeds, while limiting fatty meats, sweets, sugar-sweetened beverages, and full-fat dairy products) and healthy plant-based diet have also proven to be cardioprotective [47].

Exercise

A stable HF patient should be encouraged to engage in exercise, but it is important to consider their functional capacity [15]. According to the National Institute of Nutrition, aerobic activities such as brisk walking, jogging, and swimming increase heart rate and breathing while improving cardiovascular and lung fitness. An exercise program should include a warm-up and cool-down period, each lasting five minutes. During exercise, the intensity should ensure a 60-70% increase in heart rate [43]. According to the Physical Activity Guidelines (PAG) for Americans, adults should engage in at least 150-300 minutes a week of moderate-intensity, or 75-150 minutes a week of vigorous-intensity aerobic physical activity or an equivalent combination of moderate- and vigorous-intensity aerobic activity [48]. 

Telehealth

Telehealth improves clinical practice by promoting patient-centered care and optimizing resources. It reduces in-person outpatient visits and hospitalizations, providing an efficient healthcare experience. Emerging technologies cater to various patient needs, including complex cases like heart transplants and ventricular assist devices, ensuring timely, personalized care from home [49]. Virtual visits and forward triage can help identify patients showing signs of decompensated HF. In-hospital care can be enhanced through remote communication tools. Following discharge, patients may participate in remote follow-ups or telerehabilitation to reduce the risk of early readmissions [50].

Challenges of home-based care

Home-based care in India is becoming increasingly important but faces several significant challenges. A qualitative study involving various stakeholders identified key issues like poor interdisciplinary collaboration, where team members lack a full understanding of each other's roles, hampering effective teamwork. Additionally, there is insufficient involvement of volunteers. Social workers and medical interns, who were exposed to volunteerism in palliative care, emphasized that identifying and training volunteers would ensure uninterrupted daily care, especially during emergencies. There is a need for enhanced training, particularly in communication skills and managing emotional challenges. Both social workers and nursing staff noted that emotional concerns are often the primary focus for patients and caregivers. Finally, social workers suggested that the team should participate in emotional condolence meetings and a ritual after a patient passes away, which would strengthen social support and promote greater community participation in the program [51]. Additional challenges include inadequate infrastructure, such as insurance-related issues, difficulties with patient referrals, and the challenge of policymaking and setting regulations for home care. There are also challenges related to providing medical equipment, economic constraints like service tariffing, and resource allocation difficulties, as establishing a home care center is not cost-effective. Furthermore, there are concerns about improving the quality of home care services, including empowering human resources and monitoring the performance of home care centers [52]. An integrative review identified several key challenges in the home healthcare market for the elderly. These obstacles include a lack of standardized methods, inadequate communication, limited human resources, and cultural barriers. Furthermore, the market is underdeveloped, with high costs limiting access and jeopardizing the sustainability of public sector services [53,54]. Employee retention and utilization, operational inconsistencies in quality, a lack of standardized training protocols, and the competitive landscape characterized by disorganized players versus structured ones are significant challenges facing home-based care [6].

HF clinical care pathway

**Figure 2:**
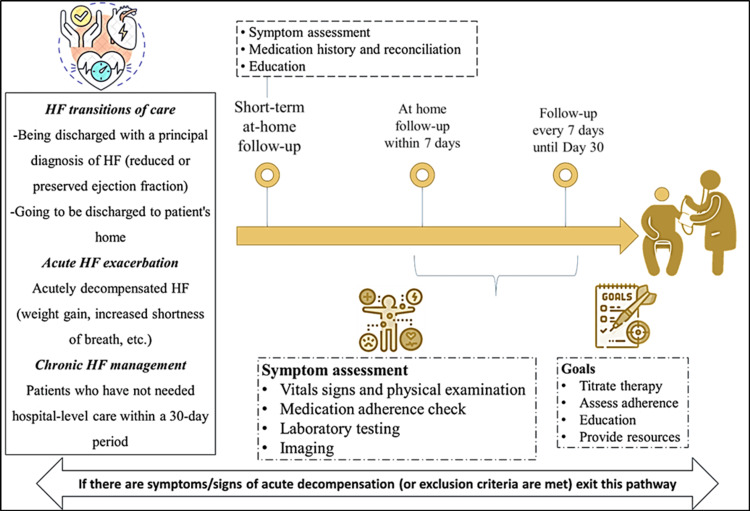
HF clinical care pathway incorporating home-based care Original figure HF: Heart failure

The HF clinical care pathway incorporates home-based care. Patients transitioning from hospital to home after a diagnosis of HF, whether acute or chronic, who do not require hospital-level care within a 30-day period, will have short-term at-home follow-ups. These follow-ups will include symptom assessments, medication history, and education. At-home follow-ups will occur within seven days of discharge and then every seven days until day 30. Each follow-up will involve symptom assessments, physical examinations, checks on medication adherence, lab testing, and imaging. The goals are to titrate therapy, evaluate adherence, manage medications, and provide necessary resources (Figure 2). Similar to this pathway, the multinational, open-label, randomized, parallel-group (STRONG-HF) trial reported that an intensive strategy involving rapid up-titration of GDMTs and close follow-up after an acute HF admission enhanced symptoms and quality of life and reduced the risk of 180-day all-cause mortality and HF readmissions [55]. The care pathway is given in detail in the workbook, which can be beneficial to both clinicians and patients.

## Conclusions

This review emphasizes the importance of post-HF home-based care in preventing recurrent cardiac events, enhancing recovery, and improving quality of life. To address the challenges of home-based care after HF, a care pathway and workbook have been designed to empower healthcare team members with essential tools and resources for delivering exceptional, patient-centered care in home settings. Ultimately, the objective is to enable seamless transitions for both patients and clinicians between traditional hospital care and innovative home-based solutions.
